# A cosolvent surfactant mechanism affects polymer collapse in miscible good solvents

**DOI:** 10.1038/s42004-020-00405-x

**Published:** 2020-11-11

**Authors:** Swaminath Bharadwaj, Divya Nayar, Cahit Dalgicdir, Nico F. A. van der Vegt

**Affiliations:** 1 Eduard-Zintl-Institut für Anorganische und Physikalische Chemie, Technische Universität Darmstadt, 64287 Darmstadt, Germany; 2 Centre for Computational and Data Sciences, Indian Institute of Technology Kharagpur, Kharagpur, West Bengal 721302 India

**Keywords:** Polymers, Chemical physics

## Abstract

The coil–globule transition of aqueous polymers is of profound significance in understanding the structure and function of responsive soft matter. In particular, the remarkable effect of amphiphilic cosolvents (e.g., alcohols) that leads to both swelling and collapse of stimuli-responsive polymers has been hotly debated in the literature, often with contradictory mechanisms proposed. Using molecular dynamics simulations, we herein demonstrate that alcohols reduce the free energy cost of creating a repulsive polymer–solvent interface via a surfactant-like mechanism which surprisingly drives polymer collapse at low alcohol concentrations. This hitherto neglected role of interfacial solvation thermodynamics is common to all coil–globule transitions, and rationalizes the experimentally observed effects of higher alcohols and polymer molecular weight on the coil-to-globule transition of thermoresponsive polymers. Polymer–(co)solvent attractive interactions reinforce or compensate this mechanism and it is this interplay which drives polymer swelling or collapse.

## Abstract (web summary)

Amphiphilic cosolvents can cause both swelling and collapse of stimuli responsive polymers, but the mechanisms governing this behavior remain unclear. Here the authors use molecular dynamics simulations to explain interfacial solvation thermodynamics, rationalizing coil-to-globule transitions of thermoresponsive polymers.

## Introduction

The polymer coil–globule transition is an intramolecular analog of a first order phase transition^1,2^. Various applications of thermoresponsive polymers dissolved in water^3^ rely on changes in material properties caused by the coil-to-globule collapse of polymer chains which occurs upon increasing the temperature. The temperature, *T*(collapse), at which this transition occurs is closely similar to the lower critical solution temperature, LCST, experimentally observed when globular chains aggregate shortly after they collapse^4,5^. Cosolvents, but also salts and small organic molecules (osmolytes) which are typically found in the living cell, affect the LCST and the free energy difference between the coil state and the globule state of various thermoresponsive polymers, such as polyacrylamides^6–11^ and elastin-like polypeptides^11,12^. It has been broadly recognized that cosolvents that partition to the polymer surface affect this free energy difference in favor of the coil state, and therefore increase polymer solubility and the LCST. By contrast, cosolvents that are depleted from the polymer surface favor the globule state over the coil state and therefore decrease polymer solubility and the LCST. Although this simple picture applies in many cases, it is not generic and a recent paradigm shift followed several intriguing observations that polymer collapse could in fact be triggered by preferential interactions with cosolvents^13–19^, specific salts^20–23^, or cosolutes^6,7,24–29^ that partition at the polymer surface.

The question we address herein is how these preferential interactions drive polymer collapse in water–alcohol mixtures. Several thermoresponsive polymers undergo coil–globule–coil transitions (cononsolvency) in binary mixtures of two good solvents (e.g., water and alcohol in the case of polyacrylamides) with increasing concentration of the cosolvent (alcohol) at a fixed temperature^4,5,14,17,30–32^. The underlying molecular mechanism is however not understood. Recently reported molecular simulations and theoretical models have emphasized the role of attractive polymer–(co)solvent interactions^5,13–15,33–35^, which can be probed in spectroscopy measurements^36,37^. A comprehensive picture is however still missing as it further requires an in-depth understanding of the role of solvent–excluded volume (repulsive) interactions in these systems. While the effect of these repulsive interactions on the polymer coil–globule collapse equilibrium has been discussed previously^16,38,39^, conclusive information remains lacking. The solvent–excluded volume contribution to the solvation free energy of a macromolecular solute corresponds to the formation of a repulsive polymer–solvent interface and is determined by the surface tension of the (mixed) solvent^40^. This contribution is not accounted for in existing polymer theories^34,35,41–45^ and cannot be modeled with an effective interaction parameter. Experimentally, its importance is reflected by the fact that the LCST of thermoresponsive polymers correlates with the surface tension increments of the aqueous salt solutions in which they are dissolved^9^.

In this work, we report molecular simulations of a generic polymer in water-alcohol mixtures and demonstrate that polymer collapse, which occurs in conjunction with preferential adsorption of amphiphilic alcohol molecules (methanol and ethanol), is driven by changes in the interface formation free energy originating from the above-described repulsive polymer–(co)solvent interactions. We demonstrate that alcohol, added to the solution at low concentration, reduces the interface formation free energy of extended coil-like chains and compact globular chains at different rates, corresponding to faster alcohol saturation and a faster lowering of the free energy of globular chains. This role of interfacial solvation thermodynamics corresponds to a surfactant mechanism driving polymer collapse. It also rationalizes experimentally observed changes in LCST behavior of higher molecular weight polymers^46,47^ and changes in LCST behavior in aqueous solutions with higher alcohols ^8,19,48^.

The surfactant mechanism proposed herein arises from solvent–excluded volume interactions with extended macromolecular surfaces. It should be generic in systems with amphiphilic cosolvents, but maybe offset or reinforced by attractive polymer–(co)solvent interactions.

## Results

### Polymer collapse free energy

**Fig. 1:**
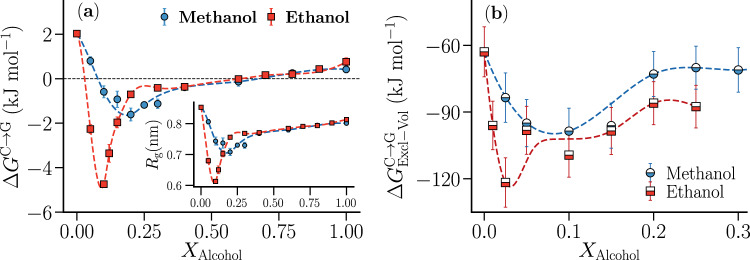
Polymer collapse free energies in water-alcohol mixtures. Dependence of **a** polymer collapse free energy *ΔG*^C→G^, radius of gyration *R*_g_ (inset) and **b** $\Delta {G}_{{\rm{Excl}}-{\rm{V}}{\rm{ol}}}^{{\rm{C}}\to {\rm{G}}}$ on the alcohol concentration *X*_Alcohol_(= *N*_Alcohol_/(*N*_Alcohol_ + *N*_Water_)). As in the case of polyacrylamides in water–alcohol mixtures^8,19,48^, the minimum in *ΔG*^C→G^ (and *R*_g_) becomes deeper and shifts to lower alcohol concentrations for the higher alcohol. Interestingly, the solvent–excluded-volume contribution, $\Delta {G}_{{\rm{Excl}}-{\rm{V}}{\rm{ol}}}^{{\rm{C}}\to {\rm{G}}}$, also shows the signature of cononsolvency and captures the effect of alcohol size, indicating its important role in driving this phenomenon. Polymer–water and polymer–alcohol Van der Waals interactions were scaled with *λ*_pw_ = 1.095 and *λ*_pa_ = 0.949, respectively (see Methods). The error bars represent the standard errors in the respective quantities.

The coil-to-globule (*C* → *G*) collapse free energy, *ΔG*^C→G^, has been derived from the two-state potential of mean force (PMF), *w*(*R*_g_), (Supplementary Figs. 1–3) using umbrella sampling with the radius of gyration, *R*_g_, as collective variable (see Methods). Figure 1 shows that the generic polymer exhibits cononsolvency where the radius of gyration *R*_g_ of the polymer (inset) and collapse free energy *ΔG*^C→G^ show a non-monotonic dependence (decrease followed by increase) on the alcohol concentration *X*_Alcohol_ = *N*_Alcohol_/(*N*_Alcohol_ + *N*_Water_), where *N*_Water_ and *N*_Alcohol_ are the number of water and alcohol molecules in the system, respectively. The minimum in *ΔG*^C→G^ decreases and shifts to lower alcohol concentration for the higher alcohol (ethanol), in agreement with the cononsolvency behavior of polyacrylamides^8,19,48^.

Interestingly, the solvent–excluded-volume contribution to the polymer collapse free energy, $\Delta {G}_{{\rm{Excl}}-{\rm{V}}{\rm{ol}}}^{{\rm{C}}\to {\rm{G}}}$, also shows a non-monotonic dependence on the alcohol concentration (Fig. 1b). This quantity, which corresponds to the difference in the reversible work of creating a polymer coil- and a polymer globule-sized cavity in solution, was obtained by thermodynamic integration employing Weeks–Chandler–Andersen (WCA)^49^ polymer–water and polymer–alcohol interactions (see Methods). Note that $\Delta {G}_{{\rm{Excl}}-{\rm{V}}{\rm{ol}}}^{{\rm{C}}\to {\rm{G}}}$ is always negative because polymer cavities with a smaller solvent-accessible-surface area (SASA) impose weaker excluded-volume restrictions on the molecules of the binary solvent. The observed non-monotonic dependence of $\Delta {G}_{{\rm{Excl}}-{\rm{V}}{\rm{ol}}}^{{\rm{C}}\to {\rm{G}}}$ on the alcohol concentration indicates that solvent–excluded-volume interactions shift the polymer coil–globule equilibrium towards the collapsed globule state upon adding a low alcohol concentration to the solution while shifting it towards the expanded coil state upon adding higher alcohol concentrations. Similar to the trends observed in *ΔG*^C→G^ and *R*_g_, the minimum in $\Delta {G}_{{\rm{Excl}}-{\rm{V}}{\rm{ol}}}^{{\rm{C}}\to {\rm{G}}}$ is deeper and shifts to lower alcohol concentration for the higher alcohol. Since $\Delta {G}_{{\rm{Excl}}-{\rm{V}}{\rm{ol}}}^{{\rm{C}}\to {\rm{G}}}$ depends only on the solute size and bulk solvent–cosolvent interactions, the results highlight the crucial role played by bulk solvent–cosolvent interactions in driving the cononsolvency phenomenon. The minimum in $\Delta {G}_{{\rm{Excl}}-{\rm{V}}{\rm{ol}}}^{{\rm{C}}\to {\rm{G}}}$ does not exactly coincide with the minimum in *ΔG*^C→G^ due to the role of attractive polymer–cosolvent interactions in determining the concentration regime of polymer collapse, as discussed later.

### Reversible work of cavity creation

**Fig. 2:**
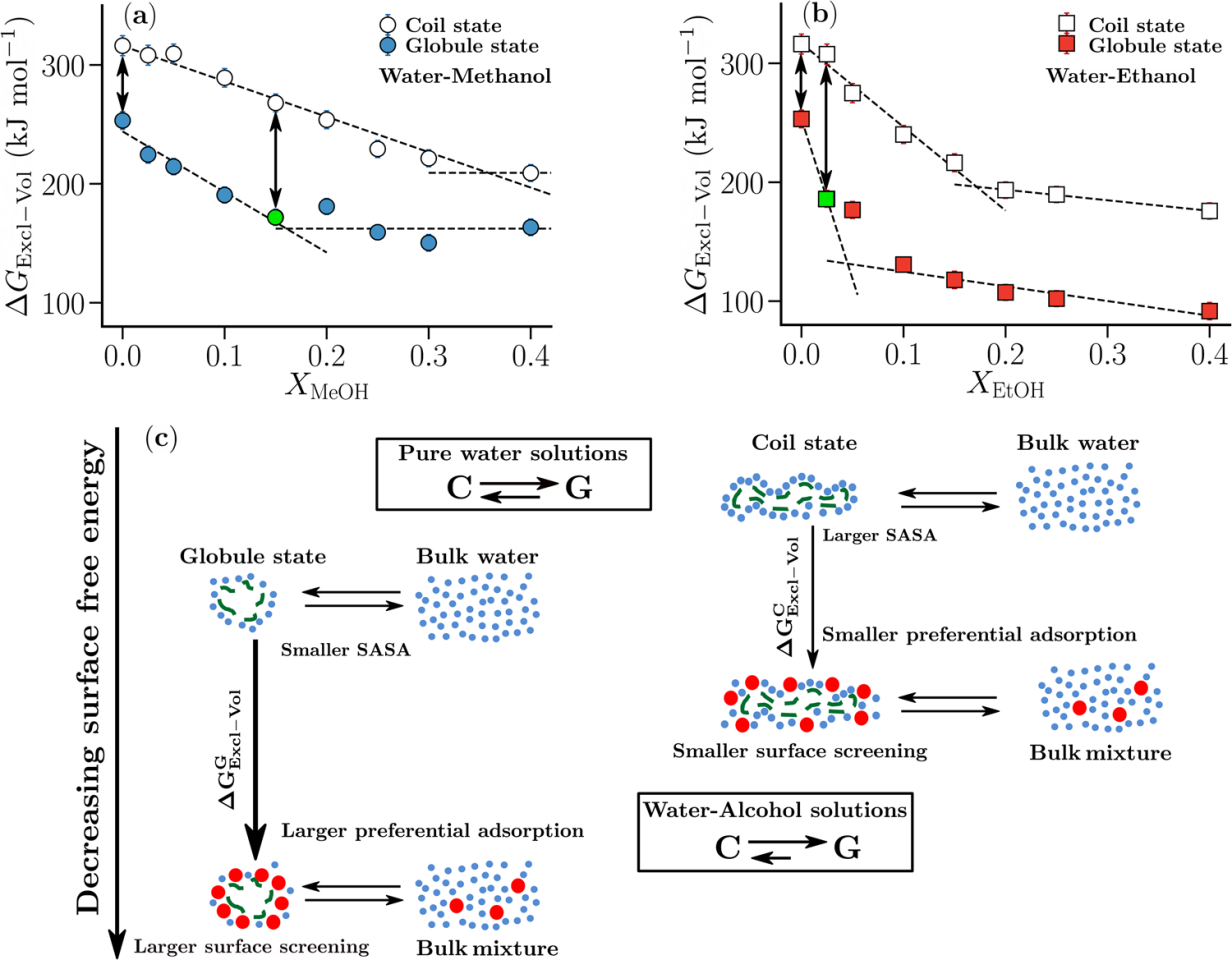
Surfactant like behavior of alcohols favors polymer collapse. Dependence of the reversible work of cavity creation $(\Delta {G}_{{\rm{Excl}}-{\rm{V}}{\rm{ol}}}^{{\rm{C}}},\Delta {G}_{{\rm{Excl}}-{\rm{V}}{\rm{ol}}}^{{\rm{G}}})$ for most probable coil and globule conformations on **a** methanol concentration *X*_MeOH_ and **b** ethanol concentrations *X*_EtOH_ for *λ*_pa_=0.949. The green markers indicate the concentrations at which the globule state is nearly saturated by methanol and ethanol, and the dashed lines are linear fits for visual aid. **c** Schematic: the trade-off between polymer surface screening and cosolvent translational entropy in bulk determines the decrease in the surface free energy, *ΔG*_Excl−Vol_, for the coil and the globule states. The globule state, due to its compact size and lower SASA, can be more readily screened by cosolvent (larger preferential adsorption) than the coil state due to which its surface free energy, $\Delta {G}_{{\rm{Excl}}-{\rm{V}}{\rm{ol}}}^{{\rm{G}}}$, decreases faster than that of the coil state ($\Delta {G}_{{\rm{Excl}}-{\rm{V}}{\rm{ol}}}^{{\rm{C}}}$). The arrows in **a** and **b** show this difference between the rates of decrease in *ΔG*_Excl−Vol_ for the coil and the globule states. The reversible work of cavity creation decreases for both states at a faster rate for water-ethanol mixtures as, for the same concentration, ethanol can screen the polymer surface more effectively due to its larger size (green marker shifts to lower alcohol concentration as one moves from methanol to ethanol). For these simulations, *λ*_pw_ = 1.095 and *λ*_pa_ = 0.949. The error bars represent the standard errors in the respective quantities.

The non-monotonic trend observed in $\Delta {G}_{{\rm{Excl}}-{\rm{V}}{\rm{ol}}}^{{\rm{C}}\to {\rm{G}}}$ correlates with the trends in enthalpy of mixing (*ΔH*_mix_) and adiabatic compressibility in pure water-alcohol mixtures as observed in the previous studies^4,8,50^. It has been proposed that adding small amounts of alcohol to a solution of a thermoresponsive polymer in neat water leads to stronger interactions in the bulk solvent which in turn increases the solvation free energy of the coil state thereby causing the polymer to collapse^4,8,44^. If this hypothesis applies, an increase in the reversible work of cavity creation of the coil state ($\Delta {G}_{{\rm{Excl}}-{\rm{V}}{\rm{ol}}}^{{\rm{C}}}$) must be observed with increasing alcohol concentration (at low concentrations) with a larger rate of increase for higher alcohols. In contrast, *ΔG*_Excl−Vol_ decreases monotonically with alcohol concentration for both coil and globule polymer states (Fig. 2a, b). These trends have also been observed for aggregates of methane molecules in water-methanol mixtures^39^. This indicates that the hypothesis involving the strengthening of interactions in the bulk mixture does not apply to amphiphilic cosolvents such as methanol and ethanol. Note that the reversible work of cavity creation decreases at a faster rate for both coil and globule states in water–ethanol (Fig. 2b) mixtures as compared to water–methanol mixtures (Fig. 2a). These trends in *ΔG*_Excl−Vol_ correlate with the trends in surface tension of water-alcohol solutions^51^, indicating that a macroscopic thermodynamic description applies in macromolecular solvation.

## Discussion

The decrease in the reversible work of cavity creation in the presence of amphiphilic cosolvents such as methanol and ethanol is caused by preferential adsorption of the cosolvent on the polymer surface (see Supplementary Figs. 4–6 in Supplementary Note 2). The corresponding screening of the hydrophobic polymer–water interface by these alcohols reduces its unfavorable interaction with water, thereby reducing the free energy of the non-polar surface. However, a loss of cosolvent translational entropy in the bulk accompanies the preferential accumulation of the cosolvent. Therefore, the extent of screening or decrease in the free energy of the non-polar surface is determined by the interplay between these two effects, which depends on the bulk cosolvent concentration. At low cosolvent concentration, the globule state, due to its compact nature and lower SASA, can be more readily screened by the cosolvent than the coil state (see schematic in Fig. 2c). This leads to a higher preferential adsorption of the alcohol to the globule state than to the expanded coil state which in turn leads to a faster decrease in $\Delta {G}_{{\rm{Excl}}-{\rm{V}}{\rm{ol}}}^{{\rm{G}}}$ (Fig. 2a, b) thereby providing the driving force that shifts the polymer coil–globule equilibrium towards the globule state. This dependence of *ΔG*_Excl−Vol_ on the alcohol concentration can also be observed in the calculations of the reversible work of cavity creation for poly(N-isopropylacrylamide) (PNIPAM) in water-methanol mixtures^16^ (see Supplementary Fig. 8 in Supplementary Note 3). Two important observations emerge from the results in Figs. 1b and 2. First, the concentration, ${X}_{{\rm{c}},\min }$, corresponding to the minimum in $\Delta {G}_{{\rm{Excl}}-{\rm{V}}{\rm{ol}}}^{{\rm{C}}\to {\rm{G}}}$, correlates with the concentration at which the globule state surface is saturated by the cosolvent (green markers in Fig. 2a, b). Second, the depth of the minimum in $\Delta {G}_{{\rm{Excl}}-{\rm{V}}{\rm{ol}}}^{{\rm{C}}\to {\rm{G}}}$ is dependent on the difference between the rates of decrease in *ΔG*_Excl−Vol_ for the coil and the globule states (arrows in Fig. 2a, b). As this difference increases, the minimum in $\Delta {G}_{{\rm{Excl}}-{\rm{V}}{\rm{ol}}}^{{\rm{C}}\to {\rm{G}}}$ becomes deeper. From Fig. 2b, it can be seen that the reversible work of cavity creation decreases, for both coil and globule states, at a faster rate in water-ethanol mixtures as compared to water–methanol mixtures. This occurs because, at the same alcohol concentration, ethanol can screen the surface more effectively than methanol due to its larger size. As a result, ${X}_{{\rm{c}},\min }$ corresponds to a lower alcohol concentration for the higher alcohol (see green markers in Fig. 2a, b). These trends correlate with the observation that the surface tension of alcohol–water mixtures decreases at a higher rate for higher alcohols^51^ and explain the cononsolvency behavior in PNIPAM–water–alcohol mixtures ^8,19,48^.

**Fig. 3:**
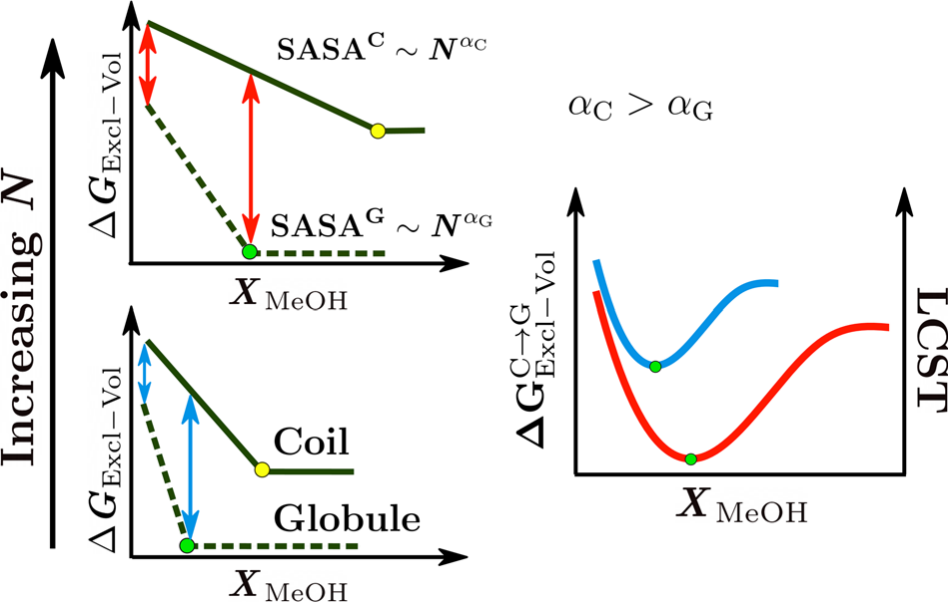
Dependence of cononsolvency on the polymer molecular weight. Schematic showing the dependence of the reversible work of cavity creation for the coil and globule states on the degree of polymerization *N* and its correlation to the LCST dependence in PNIPAM–water–methanol solutions^46,47^. The SASAs of both coil and globule states grow with increase in *N* due to which the methanol concentration required to saturate them also increases (green and yellow markers in the left panel). As the SASA of the coil state (${{\rm{SASA}}}^{{\rm{C}}} \sim {N}^{{\alpha }_{{\rm{C}}}}$) grows faster with *N* than that of the globule (${{\rm{SASA}}}^{{\rm{G}}} \sim {N}^{{\alpha }_{{\rm{G}}}}$) state, *α*_C_ > *α*_G_, the difference between the rates of decrease in *ΔG*_Excl−Vol_ for the coil and globule states rises with *N* (arrows in the left panel). These are the two aspects due to which the minimum in $\Delta {G}_{{\rm{Excl}}-{\rm{V}}{\rm{ol}}}^{{\rm{C}}\to {\rm{G}}}$, and thereby the LCST, becomes deeper and shifts to higher methanol concentration with increase in *N*. The blue and red curves in the right panel represent the dependence of $\Delta {G}_{{\rm{Excl}}-{\rm{V}}{\rm{ol}}}^{{\rm{C}}\to {\rm{G}}}$ (and LCST) on *X*_MeOH_ for the two chain lengths on the left panel.

The trends in Fig. 2 furthermore rationalize the polymer molecular weight (or degree of polymerization *N*) dependence of the cononsolvency in PNIPAM-water-methanol mixtures where the minimum in LCST becomes deeper and shifts to higher methanol concentration with increase in *N*^46,47^. For both the coil and the globule states, the SASA increases with increase in *N*, with the rate of increase being higher for the former (${{\rm{SASA}}}^{{\rm{C}}} \sim {N}^{{\alpha }_{{\rm{C}}}},{{\rm{SASA}}}^{{\rm{G}}} \sim {N}^{{\alpha }_{{\rm{G}}}},{\alpha }_{{\rm{C}}}> {\alpha }_{{\rm{G}}}$, see Supplementary Note 4). Due to the larger SASA, a higher cosolvent concentration is required for saturating the globular (and coiled) surface which in turn leads to an increase in ${X}_{{\rm{c}},\min }$ (green markers in Fig. 3). Further, the difference between the rates of decrease in *ΔG*_Excl−Vol_ for the coil and globule states rises with *N* (arrows in Fig. 3), as the SASA of the former grows (with *N*) faster than the latter, due to which the minimum in $\Delta {G}_{{\rm{Excl}}-{\rm{V}}{\rm{ol}}}^{{\rm{C}}\to {\rm{G}}}$ becomes deeper. Therefore, the minimum in $\Delta {G}_{{\rm{Excl}}-{\rm{V}}{\rm{ol}}}^{{\rm{C}}\to {\rm{G}}}$, and in turn the LCST, becomes deeper and shifts to higher cosolvent concentration with increase in the molecular weight (right panel of Fig. 3). Note that one would expect similar trends with increase in the size of the monomer as well.

**Fig. 4:**
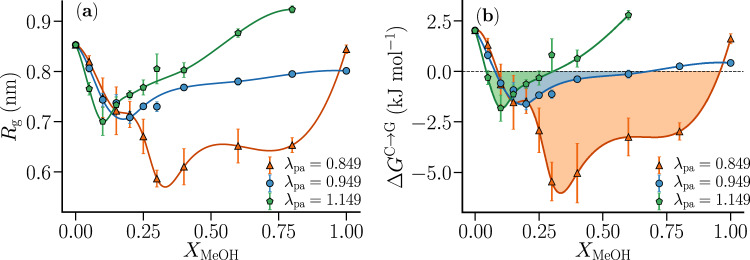
Role of polymer–methanol attractive interactions in cononsolvency. Dependence of **a** radius of gyration *R*_g_, and **b** polymer collapse free energy *ΔG*^C→G^, on the methanol concentration *X*_MeOH_ for different *λ*_pa_ values. The concentration range over which the globule is thermodynamically favored, *ΔG*^C→G^ < 0, decreases with increase in the polymer–methanol attractive strength. It is expected that the preferential adsorption of methanol on the polymer would increase as the polymer–methanol attractive interaction strengthens. This increasing preferential adsorption shifts the polymer coil–globule equilibrium towards the coil state. This shows that preferential adsorption can favor either swelling or collapse of the polymer depending on the underlying microscopic interactions. For the above calculations *λ*_pw_ = 1.095. The error bars represent the standard errors in the respective quantities.

Interestingly, we find that the solvent–excluded volume of the polymer is not the only factor that determines the polymer collapse. The attractive polymer–cosolvent interactions are also found to play a crucial role, as reflected in the shift of the minima in *ΔG*^C→G^ (and *R*_g_) as a function of the strength of the polymer–alcohol attractions (see Fig. 4). Note that $\Delta {G}_{{\rm{Excl}}-{\rm{V}}{\rm{ol}}}^{{\rm{C}}\to {\rm{G}}}$ is very weakly dependent on *λ*_pa_ as the polymer interacts with the solvent/cosolvent mixture through the WCA potential (see Supplementary Fig. 10 in Supplementary Note 5). Strengthening of the polymer–methanol attractive interactions (increasing *λ*_pa_) shifts the polymer coil–globule equilibrium towards the expanded coil state, thereby reducing the concentration range where the collapsed state is thermodynamically favorable, *ΔG*^C→G^ < 0 (shaded regions in Fig. 4b). We attribute this trend to be the reason behind the experimentally observed absence of cononsolvency in poly(N,N-diethylacrylamide) (PDEA)–water–methanol mixtures^14,19,31,52^. Since PDEA has a larger non-polar surface area than PNIPAM^28^, the PDEA–methanol van der Waals interaction (relative to PDEA–water interaction) would be stronger than PNIPAM–methanol interaction (relative to PNIPAM–water interaction). For a fixed polymer–water interaction strength (*λ*_pw_ = 1.095), it is expected that strengthening of the polymer–methanol attractive strength (increase in *λ*_pa_) would lead to an increase in the preferential adsorption of methanol. This preferential adsorption, driven by the stronger polymer–cosolvent attractive interactions, shifts the polymer coil–globule equilibrium towards the coil state in the system studied herein (see Fig. 4). On the other hand, the preferential adsorption on the repulsive polymer surface due to the surfactant-like behavior of amphiphilic cosolvents (Fig. 1b) shifts the polymer coil–globule equilibrium towards the globule state. From Supplementary Figs. 4-7, it can be seen that the extent of preferential alcohol adsorption on the repulsive polymer surface (surfactant like behavior) is higher than that of the fully interacting polymer (see Supplementary Note 2 for more details). This clearly shows the prominent role played by the excluded volume interactions in driving the preferential accumulation of the cosolvent onto the polymer surface. We note that for strongly interacting co-solutes such as guanidinium thiocyanate salt and urea, attractive interactions lead to polymer collapse^7,13,20,28,53^. Therefore, depending on the underlying microscopic interactions, preferential cosolvent adsorption can either lead to polymer collapse or expansion. This may be the reason for the inability of generic models^15,54^, based only on preferential adsorption, to explain the differences between the behavior in PNIPAM and PDEA solutions. These observations indicate that cononsolvency depends on the interplay between the solvent–excluded-volume interactions, originating from bulk solvent–cosolvent interactions^8,44,55^, and the polymer–solvent/cosolvent attractive interactions^5,13,14,33,34,41^.

In conclusion, we showed that the polymer coil–globule equilibrium is governed by the interplay of solvent–excluded-volume interactions (free energy cost of creating a repulsive polymer–solvent interface) and polymer–solvent/cosolvent attractive van der Waals interactions. Our results demonstrate that amphiphilic cosolvents, such as methanol and ethanol, reduce the free energy cost of creating a repulsive polymer–solvent interface through a surfactant mechanism which surprisingly shifts the coil–globule equilibrium towards the collapsed globule state at low alcohol concentrations. The surfactant mechanism found herein is generic and should have important consequences for the physical properties of a wide variety of macromolecular systems. This is evident from the observation that the proposed interplay is able to rationalize the cononsolvency phenomenon of acrylamide polymers, and its dependence on the cosolvent size and polymer molecular weight.

## Methods

### System details

We used a generic hydrophobic polymer model developed by Zangi et al.^56^ consisting of 32 uncharged Lennard-Jones beads with *σ*_p_ =  0.4 nm and *ϵ*_p_ = 1.0 kJ mol^−1^. The angular and bonded force-field parameters were taken from the same reference^56^. The aqueous polymer solution consisted of 5000 SPC/E^57^ water molecules in a cubic box and the aqueous alcohol polymer solutions were described with the OPLS-UA force-field for methanol and ethanol (5000 molecule water-alcohol mixture)^58^. The generic polymer used in this work features a two-state conformational equilibrium C  ⇄  G, between coil (C) and globule (G) states^56^. Investigating cononsolvency for such a polymer model circumvents the sampling bottlenecks associated with atomistic models of real polymer systems such as of PNIPAM^33,59–61^. Our previous work showed that this model represents a poor solvent condition at 300 K and 1 atm, i.e., it provides a negative polymer collapse free energy of approximately  −2 kJ mol^−1^ in SPC/E water^62^. To achieve good solvent conditions, the polymer–solvent interaction parameters (polymer–alcohol, *ϵ*_pa_ and polymer–water, *ϵ*_pw_) were tuned while keeping the Lennard–Jones diameters (*σ*_pa_ and *σ*_pw_) unchanged compared to the original model. The unlike interactions were described with Lorentz–Berthelot mixing rules. The polymer–solvent/cosolvent interaction parameter was scaled using a parameter *λ*_px_, such that the ${\epsilon }_{{\rm{px}}}^{{\rm{new}}}={\lambda }_{{\rm{px}}}{\epsilon }_{{\rm{px}}}$, where *x* is either alcohol (a) or water (w). The *λ*_px_ values were tuned to achieve positive collapse free energies in pure water and pure alcohol (methanol and ethanol) systems (see Supplementary Figs. 1–3). The *λ*_px_ values used for the pure solvent systems and the alcohol-water mixtures were *λ*_pw_ = 1.095 for water, and *λ*_pa_ = 0.949 for methanol and ethanol. To study the effect of polymer–cosolvent interaction parameters, additional simulations were performed with *λ*_pa_ = 0.849, 1.149 for the polymer in water–methanol solutions.

### Umbrella Sampling

Potential of mean force (PMF) profiles, *w*(*R*_g_), of the polymer in different methanol–water and ethanol–water mixtures were computed by carrying out umbrella sampling simulations with the GROMACS (version 4.6.7) package^63^ using the PLUMED 2.2.0 plugin^64^ and the polymer radius of gyration (*R*_g_) as the collective variable. The harmonic restrain potential, *V*(*R*_g_), applied on the radius of gyration has the following form,

1 $$V({R}_{{\rm{g}}})=\frac{{k}_{{\rm{b}}}}{2}{\left({R}_{{\rm{g}}}-{R}_{{\rm{g}}}^{{\rm{o}}}\right)}^{2},$$

where *k*_b_(= 20000 kJ mol^−1^nm^2^) is the force constant and ${R}_{{\rm{g}}}^{{\rm{o}}}$ is the desired value of the radius of gyration. The collapsed and extended conformations of the polymer were determined using the two distinct minima in the PMF profiles below and above ${R}_{{\rm{g}}}^{\#}=$ 0.7 nm, respectively. Polymer conformations were sampled for *R*_g_ values between 0.4 nm and 1.2 nm with a spacing of *ΔR*_g_ = 0.025 nm between successive windows. The equilibration and production runs for each window were performed in the NPT ensemble for 1 ns and 20 ns, respectively. For both the equilibration and production runs, the Nosé–Hoover thermostat^65,66^ (*τ*_*T*_ =  0.5 ps) and Parrinello–Rahman barostat^67^ (*τ*_*P*_ =  1 ps) were used. A 2 fs time step was used for the integration of the equations of motion. The Van der Waals interactions were truncated at 1.4 nm. Long-range Coulombic interactions were calculated with Particle Mesh Ewald^68^ using a 1.4 nm real-space cutoff and a 0.12 nm grid spacing. The unbiased PMF was obtained using the weighted histogram analysis method (WHAM)^69^. The free energy change on polymer collapse, *ΔG*^C→G^ was computed using,

2 $${e}^{-\Delta {G}^{{\rm{C}}\to {\rm{G}}}/RT}=\frac{\mathop{\int}\nolimits_{0}^{{R}_{{\rm{g}}}^{\#}}{e}^{-w({R}_{{\rm{g}}})/RT}d{R}_{{\rm{g}}}}{\mathop{\int}\nolimits_{{R}_{{\rm{g}}}^{\#}}^{\infty }{e}^{-w({R}_{{\rm{g}}})/RT}d{R}_{{\rm{g}}}}$$

where *R* is the gas constant, *T* is the temperature and *R*_g_ is the radius of gyration, ${R}_{{\rm{g}}}^{\#}$= 0.7 nm is the cutoff for the *R*_g_ to determine the coil and the globule states of the polymer.

### Thermodynamic integration

The reversible work of cavity formation for the coil and globule states was calculated using the thermodynamic integration (TI) method implemented in Gromacs (version 2019.3)^63^ at different alcohol (methanol and ethanol) concentrations on the most probable coil (*R*_g_ = 1.0 nm) and globule (*R*_g_ = 0.5 nm) configurations. Position restraints, involving a harmonic potential with a force constant of 10^5 ^kJ mol^−1^ nm^−2^, were applied on each polymer atom to keep the chain conformations fixed. In these TI calculations, polymer–water and polymer–alcohol WCA interactions^49^ were slowly introduced. A leapfrog stochastic dynamics integrator^70^ with an inverse friction constant of 0.1 ps was used for integrating the Newton’s equations of motion and for maintaining the temperature of the system at 300 K. A total of 21 *λ* values with a spacing of 0.05 for 0 ≤ *λ* ≤ 1.0 were used. The state *λ* = 0 corresponds to the cavity-free binary solvent. A soft-core potential with soft-core parameters *α* = 0.5, *p* = 1 and *σ* = 0.3 nm was used to avoid singularities at the end state^71^. For each *λ* value, the energy of the system was minimized using the steepest descent algorithm. The equilibration process consisted of a 50 ps NVT run followed by a 100 ps NPT simulation (Berendsen barostat^72^*, τ*_p_=1 ps). This was followed by a 5 ns production run (Parrinello–Rahman barostat, *τ*_p_=1 ps) in which the first 200 ps were discarded.

## Supplementary information

Supplementary Information

## Data Availability

Data is available from the authors upon reasonable request.
