# Supplementary material for: A cosolvent surfactant mechanism affects polymer collapse in miscible good solvents
Source: Commun Chem. 2020 Nov 11;3:165. doi: 10.1038/s42004-020-00405-x (PMC9814688; doi:10.1038/s42004-020-00405-x)
Supplement: Supplementary file 1 — Supplementary Information [file 42004_2020_405_MOESM1_ESM.pdf]

Supporting Information
A cosolvent surfactant mechanism affects polymer collapse in miscible good solvents

Swaminath Bharadwaj[1*†], Divya Nayar[1,2†], Cahit Dalgicdir[1†], and Nico F. A. van der Vegt[1*]

[1]Eduard-Zintl-Institut für Anorganische und Physikalische Chemie, Technische Universität Darmstadt, 64287 Darmstadt, Germany
[2]Centre for Computational and Data Sciences, Indian Institute of Technology Kharagpur, West Bengal-721302, India
[*]email: bharadwaj@cpc.tu-darmstadt.de; vandervegt@cpc.tu-darmstadt.de
[†]These authors contributed equally

This document includes:

- Coil-to-globule transitions in aqueous alcohol solutions of a generic polymer
- Preferential binding coefficients
- Cavity contribution in PNIPAM-water-methanol solutions
- Dependence of the SASA on the molecular weight
- Effect of $\lambda_{\rm pa}$ on $\Delta G^{\rm C \to G}_{\rm Excl-Vol}$
- References

Supplementary Note 1

Coil-to-globule transitions in aqueous alcohol solutions of a generic polymer

The generic polymer model studied in this work shows two main peaks in the probability density distribution of the radius of gyration. This two-state behavior is characteristic for a system with a first order coil-globule transition in which the coil state ensemble and globule state ensemble coexist. Recent theoretical studies have shown that a generic interacting self-avoiding chain exhibits a first order coil-to-globule transition when the contributions from vibrational entropy are taken into account.[1] Experimental DSC measurements[2] have shown that poly(N-isopropylacrylamide) (PNIPAM) exhibits a first order transition (two state behavior) in aqueous solutions. Additionally, recent simulations studies involving replica exchange molecular dynamics simulations[3] and metadynamics simulations[4] show that this two-state behavior is applicable for short PNIPAM chains (30-40mers) as well.

In this study, we employ the 32mer generic polymer developed by Zangi et al.[5] which exhibits a two-state conformational equilibrium C⇄G, between the coil and globule states. Investigating cononsolvency for such a polymer model circumvents the challenges associated with obtaining sufficient conformational sampling that have been observed for atomistic models of real polymer systems such as of PNIPAM.[6] The sampling bottlenecks associated with capturing reversible transitions C⇄G of PNIPAM, occurring on typical time scales of 100 ns, can therefore be avoided.[6–9]

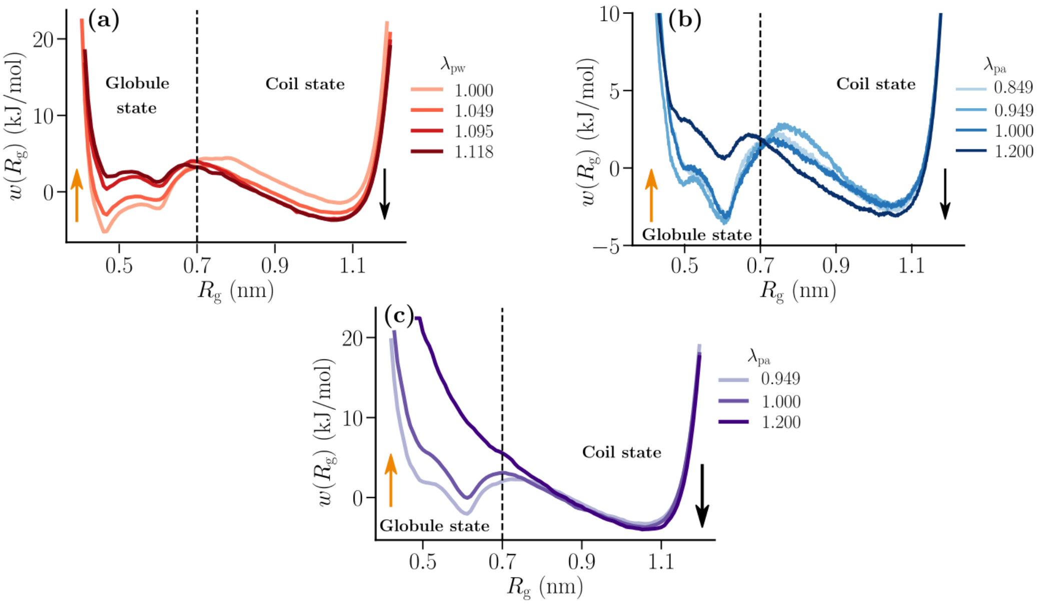

Supplementary Figure 1: Normalized PMF profiles, $w(R_g)$, for a generic polymer in (a) pure water solution at different λ_{pw} values, (b) pure methanol solutions at different λ_{pa} values and (c) pure ethanol solutions at $\lambda_{pa} = 0.949, 1.0, 1.2$. The black dashed line ($R_g^{\#} = 0.7$ nm) is the cutoff value which separates the coil and globule state regimes. The orange (black) arrow shows that the globule (coil) state becomes progressively less (more) favorable with increase in λ_{pw} (or λ_{pa}), i.e. the polymer becomes more hydrophilic (or solvophilic). The presence of two distinct minima at all conditions (hydrophilic or hydrophobic) indicates that the generic polymer, similar to acrylamide polymers,[2–4] exhibits a two-state behavior in aqueous alcohol solutions.

From the potential of mean force (PMF) profiles, $w(R_g)$, in Fig. 1, it can be seen that generic polymer shows a two-state behavior with clearly distinct minima in pure water and pure alcohol solutions. The cutoff value, $R_g^{\#}$, of the radius of gyration separating the coil and globule states corresponds to $R_g^{\#} = 0.7$ nm (black dashed line in Fig. 1). The polymer collapse free energy, $\Delta G^{C\rightarrow G} (= \Delta G^{G} - \Delta G^{C})$, is then calculated from the PMF profiles using the following expression,

$$e^{-\Delta G^{C\rightarrow G}/RT} = \frac{\int_0^{R_g^{\#}} e^{-w(R_g)/RT} \mathrm{d}R_g}{\int_{R_g^{\#}}^{\infty} e^{-w(R_g)/RT} \mathrm{d}R_g} \tag{1}$$

where R is the gas constant and T is the temperature. The PMF is normalized such that

$$\int_0^\infty e^{-w(R_\mathrm{g})/RT} \mathrm{d}R_\mathrm{g} = 1. \tag{2}$$

Given this normalization, one can define the probability distribution function $\rho(R_\mathrm{g})$ in the following way,

$$\rho(R_\mathrm{g}) = e^{-w(R_\mathrm{g})/RT}. \tag{3}$$

The two-state behavior can be suitably influenced by tuning the polymer-solvent interaction energies (polymer-alcohol, ϵ_pa, and polymer-water, ϵ_pw). The unlike interactions were described using Lorentz-Berthelot mixing rules. The polymer-water (polymer-alcohol) interaction energy was scaled using a parameter $\lambda_\mathrm{pw}(\lambda_\mathrm{pa})$, such that $\epsilon_\mathrm{pw}^\mathrm{new} = \lambda_\mathrm{pw}\epsilon_\mathrm{pw}^\mathrm{old}$ ($\epsilon_\mathrm{pa}^\mathrm{new} = \lambda_\mathrm{pa}\epsilon_\mathrm{pa}^\mathrm{old}$). From Fig. 1, it can be observed that minimum in the PMF for the coil (globule) state decreases (increases) with increase in λ_pw or λ_pa, i.e. the polymer becomes progressively more hydrophobic or solvophilic. For the original model of Zangi et al.[5] ($\lambda_\mathrm{pw} = 1$ in Fig. 1(a)), SPC/E water is a poor solvent resulting in a negative collapse free energy. In this study, we fix $\lambda_\mathrm{pw} = 1.095$ which ensures a positive collapse free energy.

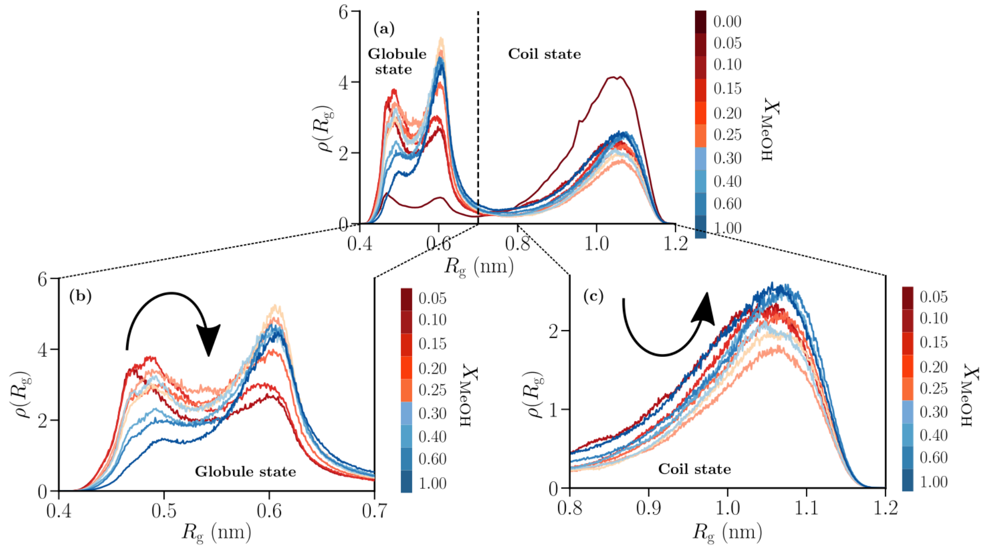

Supplementary Figure 2: (a) Profiles of the probability distribution $\rho(R_\mathrm{g})$ for different methanol concentrations at ($\lambda_\mathrm{pw} = 1.095, \lambda_\mathrm{pa} = 0.949$). The dashed line ($R_\mathrm{g}^\# = 0.7$) indicates the cutoff value which separates the coil and globule regions. A magnified view of the trends in the globule and coil state is shown in (b) and (c), respectively. The arrows in (b) and (c) show the dependence of $\rho(R_\mathrm{g})$, in the respective regions, on the methanol concentration. $\rho(R_\mathrm{g})$ shows a non-monotonic, though opposite, dependence on X_MeOH in the coil (decrease followed by increase) and globule region (increase followed by decrease).

Figure 2 shows the profiles of the probability distribution $\rho(R_\mathrm{g})$ for different methanol concentrations. The polymer solution exhibits cononsolvency as $\rho(R_\mathrm{g})$ in the globule (coil) state in Fig. 2(b) (Fig. 2(c)) shows a non-monotonic increase followed by decrease (decrease followed by increase), dependence on the methanol concentration. Similar trends are observed for the polymer in water-ethanol mixtures as well (see Fig. 3).

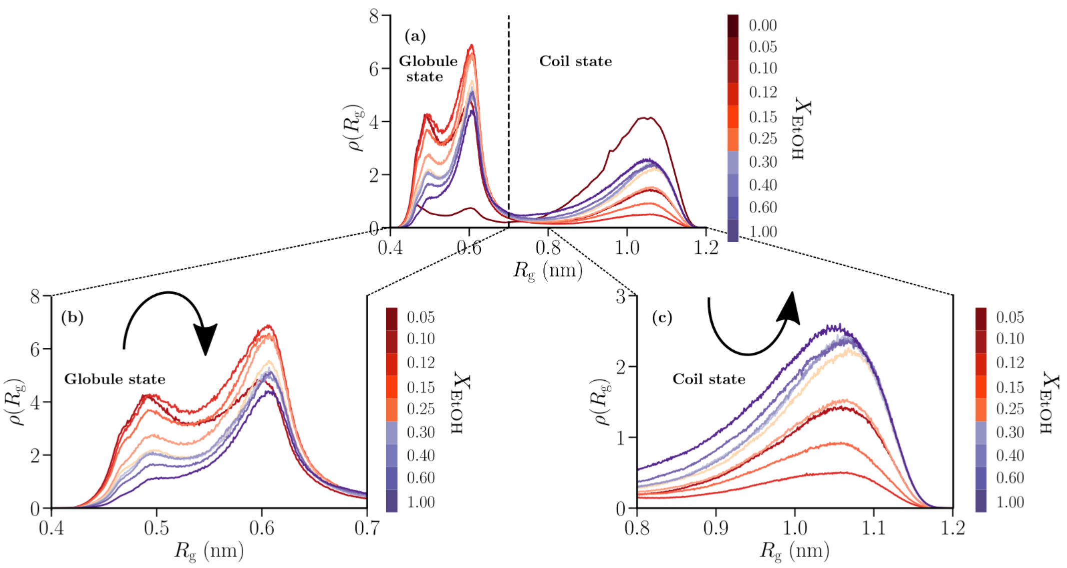

Supplementary Figure 3: (a) Profiles of the probability distribution $\rho(R_g)$ for different ethanol concentrations at ($\lambda_{pw} = 1.095, \lambda_{pa} = 0.949$). The dashed line ($R_g^{\#} = 0.7$) indicates the cutoff value which separates the coil and globule regions. A magnified view of the trends in the globule and coil states is shown in (b) and (c), respectively. The arrows in (b) and (c) show the dependence of $\rho(R_g)$, in the respective regions, on the methanol concentration. $\rho(R_g)$ shows a non-monotonic, though opposite, dependence on X_{EtOH} in the coil (decrease followed by increase) and globule region (increase followed by decrease).

Supplementary Note 2

Preferential binding coefficients

The preferential adsorption of the alcohol on the polymer chain was quantified through the preferential adsorption coefficient Γ_{23} which is the excess number of cosolvent molecules with respect to the excess number of solvent molecules in the hydration shell of the polymer and is given by the following expression,

$$\Gamma_{23} = \rho_3 \left(G_{23} - G_{21} \right), \tag{4}$$

where the index $i = 1$ stands for water, $i = 2$ for the polymer and $i = 3$ for alcohol. ρ_3 is the number density of the alcohol and G_{23} (G_{21}) is the polymer-alcohol (polymer-water) Kirkwood-Buff Integral. The cosolvent molecules are preferentially adsorbed (depleted) for $\Gamma_{23} > 0$ ($\Gamma_{23} < 0$). Γ_{23} can also be expressed in terms of number of solvent and cosolvent molecules as,

$$\Gamma_{23} = \left\langle n_3(r) - \frac{N_3 - n_3}{N_1 - n_1} n_1(r) \right\rangle, \tag{5}$$

where $n_x(r)$ denotes the number of water or alcohol molecules within a proximal distance of r from the polymer surface, and N_x, the total number of water or alcohol molecules in the system. Here, the preferential adsorption coefficients were calculated using Eq. 5. The preferential binding coefficients were calculated from a production run of 50 ns. The trajectory was divided into 10 blocks of 5 ns each to obtain 10 averaged $\Gamma_{23}(r)$. The associated errors were calculated accordingly. From these 10 averages, the final $\Gamma_{23}(r)$ computed where the errors were obtained using the Gaussian error propagation method.

Figure 4 shows the dependence of $\Gamma_{23}(r)$ on the proximal distance from the polymer, r for the coil and the globule states at different methanol concentrations for the fully interacting and repulsive polymers. The thermodynamic limiting value of Γ_{23} (Figure 4(a)) was obtained by averaging over a proximal distance of 1.0-2.0 nm for the fully interacting polymer and 1.0-1.5 nm for the repulsive polymer (Figure 4(b)) and the corresponding error was calculated using Gaussian error propagation. The dependence of the thermodynamic limiting value of Γ_{23} on the methanol concentration for the fully interacting polymer and the repulsive polymer can be seen in Fig. 5. Note that Γ_{23} is larger for the repulsive polymer in comparison to the fully interacting polymer. This clearly shows the prominent role played by the excluded volume interactions in driving the preferential accumulation of methanol molecules on to the polymer surface. Similar trends can be seen for the polymer in

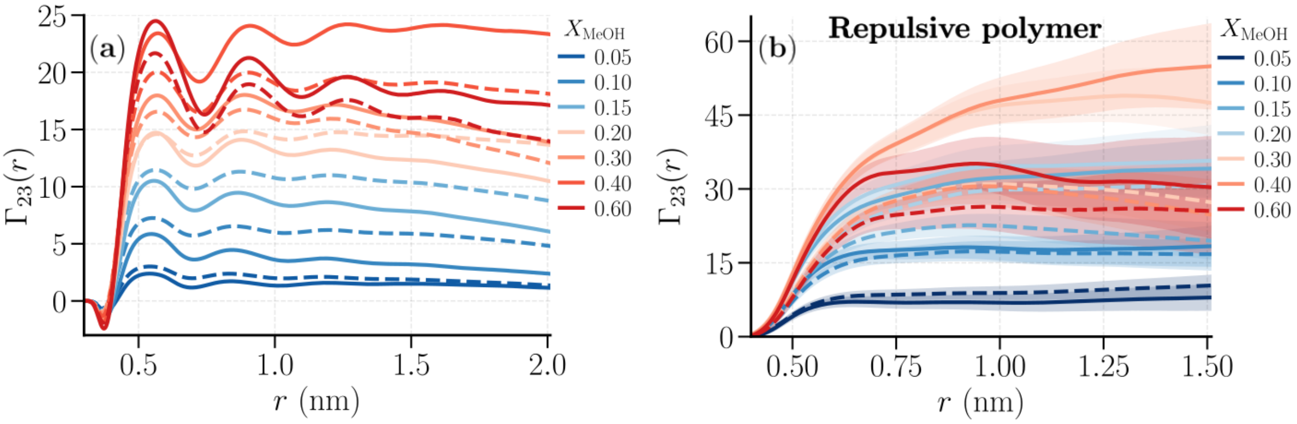

Supplementary Figure 4: Dependence of the preferential binding coefficient $\Gamma_{23}(r)$ on the proximal distance from the polymer, r, for the coil (solid lines) and the globule (dashed lines) states at different methanol concentrations for (a) fully interacting polymer and (b) repulsive polymer.

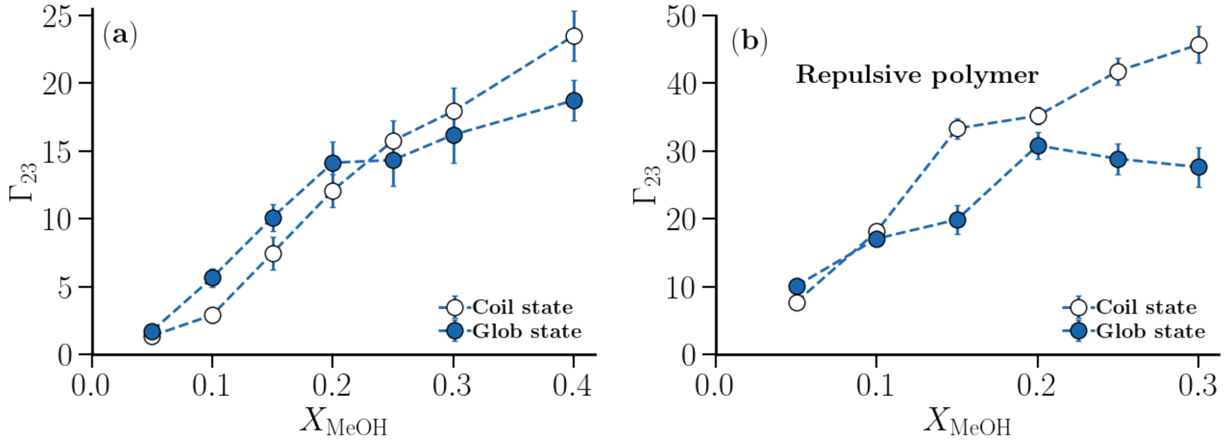

Supplementary Figure 5: Dependence of the preferential binding coefficient Γ_{23} (thermodynamic limiting value), for the coil and the globule states on the methanol concentration for (a) fully interacting polymer and (b) repulsive polymer.

water-ethanol solutions as well (see Fig. 6). The representative simulation snapshots in Fig. 7 also show the prominent role played by the excluded volume interactions in driving preferential cosolvent accumulation.

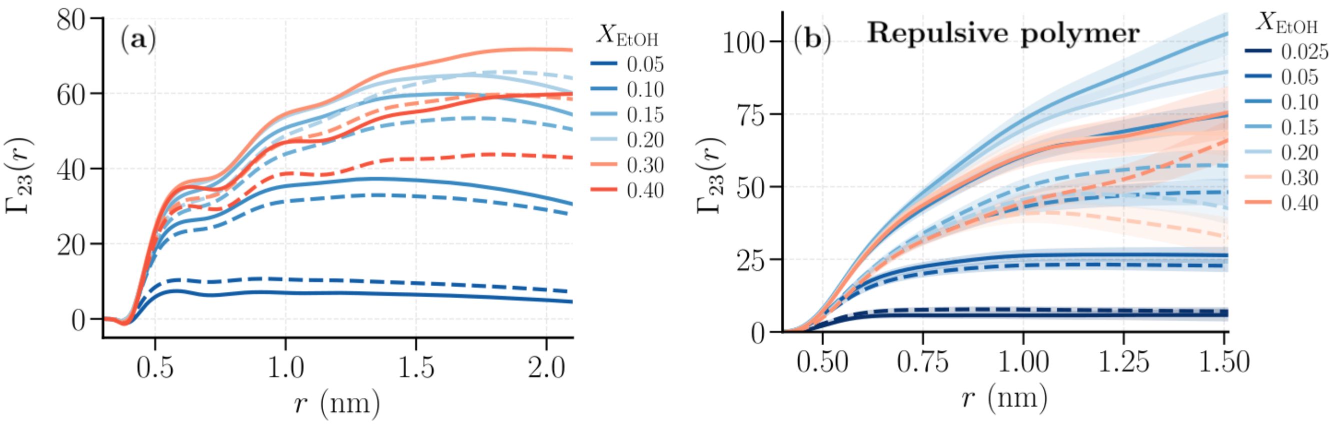

Supplementary Figure 6: Dependence of the preferential binding coefficient $\Gamma_{23}(r)$ on the proximal distance from the polymer, r, for the coil (solid lines) and the globule (dashed lines) states at different ethanol concentrations for (a) fully interacting polymer and (b) repulsive polymer.

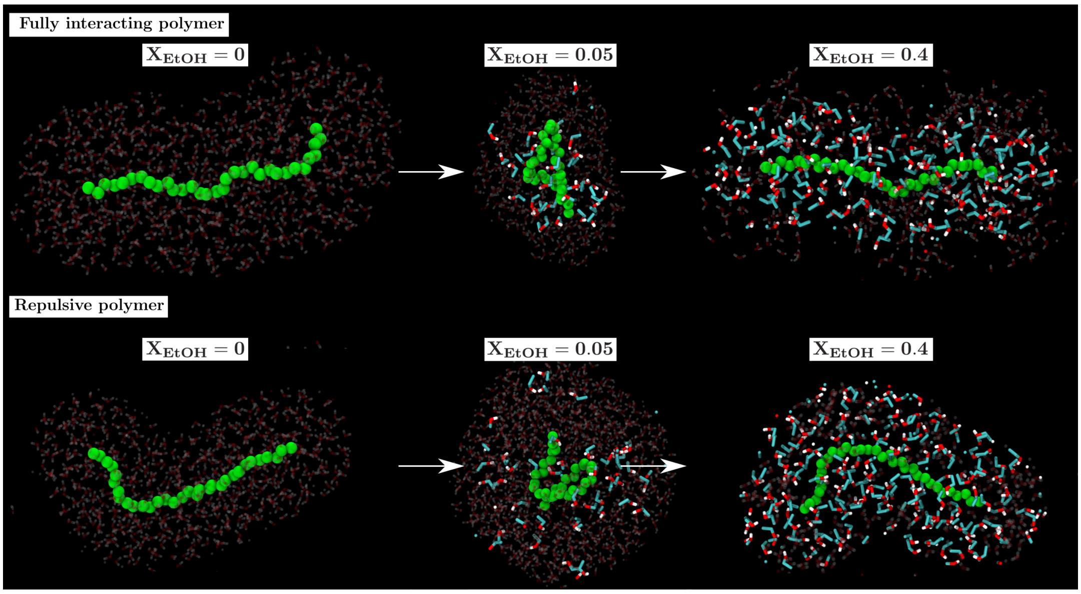

Supplementary Figure 7: Top panel shows the representative simulation snapshots for the fully interacting polymer at different ethanol concentrations. The bottom panel shows the snapshots for these states for the repulsive polymer at the same ethanol concentrations.

Supplementary Note 3

Cavity contribution in PNIPAM-water-methanol solutions

Our results show that the reversible work of cavity creation, for both the coil and the globule states, decreases with increase in the alcohol concentration (low alcohol concentration) via a surfactant like mechanism (Figure 2 in main text). We observe that the concentration $X_{\rm c,min}$, corresponding to the minimum in $\Delta G^{\rm C\rightarrow G}_{\rm Excl-Vol}$, correlates with the concentration at which the globule state surface is saturated by the alcohol. Further, the depth of the minimum in $\Delta G^{\rm C\rightarrow G}_{\rm Excl-Vol}$ is dependent on the difference between the rates of decrease in $\Delta G_{\rm Excl-Vol}$ for the coil and the globule states. Interestingly, similar trends have been observed in the simulation study by Ropero and Van der Vegt [10] on PNIPAM-water-methanol solutions. Figure. 8(a) shows the reversible work of cavity creation for the representative coil and globule states in PNIPAM-water-methanol solutions (see ref [10] for simulation details). The reversible work of cavity creation decreases, for both the coil and the globule states, with increase in the methanol concentration. Additionally, the concentration at which the globule state

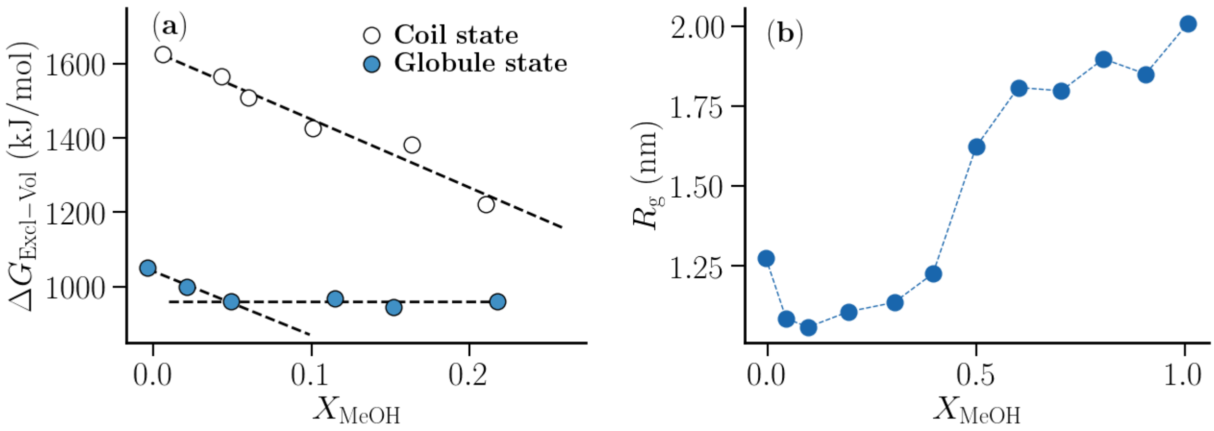

Supplementary Figure 8: Dependence of (a) reversible work of cavity creation of representative coil and globule states of PNIPAM and (b) radius of gyration R_{g}, on methanol concentration X_{MeOH}. The data for both plots is taken from ref[10].

surface is almost saturated with methanol ($X_{\text{MeOH}} \approx 0.05$) correlates with the concentration corresponding to the minimum in the radius of gyration R_{g} (see Fig. 8(b)). Note that the generic polymer shows a significant difference between the rates of decrease in $\Delta G_{\text{Excl-Vol}}$ for the coil and globule states (Figure 2(a) in main text), which seems to be absent in Fig. 8(a). The reason behind this may be that the re-entrant transition (increase in R_{g}) commences from the lowest alcohol concentration, $X_{\text{MeOH}} \approx 0.05$, itself. Given that the lowest probed concentration correlates with the concentration at which the globule state is saturated with methanol, one may have to perform simulations at much lower concentrations to observe the faster decrease in $\Delta G^{\text{G}}_{\text{Excl-Vol}}$. These results indicate that the surfactant mechanism is able to rationalize the trends in real polymer systems which further highlights its generic nature.

Supplementary Note 4

Dependence of the SASA on the molecular weight

Our results show that the reversible work of cavity creation of the globule state ($\Delta G^{\mathrm{G}}_{\mathrm{Excl-Vol}}$) decreases faster than the coil state ($\Delta G^{\mathrm{C}}_{\mathrm{Excl-Vol}}$) with increase in the alcohol concentration (at low alcohol concentrations). The difference between the rates of decrease in $\Delta G_{\mathrm{Excl-Vol}}$ for the globule and coil states, which determines the depth of the minimum in $\Delta G^{\mathrm{C}\to\mathrm{G}}_{\mathrm{Excl-Vol}}$, correlates with the corresponding difference between the SASA of the two states. Figure 9 shows the dependence of the SASA of the coil and the globule states on the degree of polymerization N. As the coil state has an extended conformation (with very few polymer-polymer contacts), an increase in N leads to a proportional increase in SASA, i.e. $\mathrm{SASA}^{\mathrm{C}} \sim N$. The globule state, on the other hand, has a compact spherical shape (with many polymer-polymer contacts) (see Fig. 9). Then, the SASA is dependent on the radius of gyration R_{g}, through $\mathrm{SASA}^{\mathrm{G}} \sim R_{\mathrm{g}}^2$. An increase in N leads to an increase in the radius of gyration ($R_{\mathrm{g}} \sim N^{1/3}$) which in turn increases the SASA, i.e. $\mathrm{SASA}^{\mathrm{G}} \sim N^{2/3}$. As $\mathrm{SASA}^{\mathrm{C}}$ grows faster with N than $\mathrm{SASA}^{\mathrm{G}}$, the difference between the rates of decrease in $\Delta G_{\mathrm{Excl-Vol}}$ for the coil and the globule states rises with N. This in turn leads to the minimum in $\Delta G^{\mathrm{C}\to\mathrm{G}}_{\mathrm{Excl-Vol}}$ which becomes deeper with increase in N.

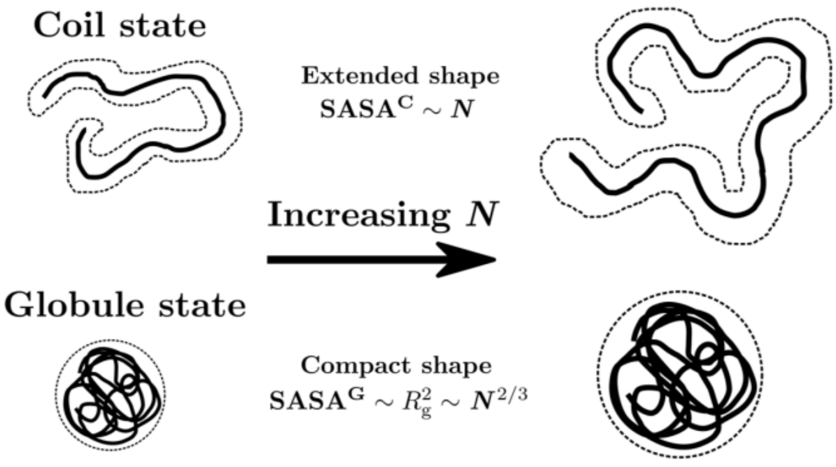

Supplementary Figure 9: Schematic showing the scaling of the SASA (dashed lines) with the degree of polymerization N for the coil and the globule states. The coil state has an extended conformation, with minimal polymer-polymer contacts, due to which an increase in N leads to a proportional increase in $\mathrm{SASA}^{\mathrm{C}}$. On the other hand the SASA of the globule state, due its compact shape (high polymer-polymer contacts), can be related to the radius of gyration through $\mathrm{SASA}^{\mathrm{G}} \sim R_{\mathrm{g}}^2$. With increase in the N, the radius of gyration increases ($R_{\mathrm{g}} \sim N^{1/3}$) which in turn increases the SASA of the globule state, $\mathrm{SASA}^{\mathrm{G}} \sim N^{2/3}$.

The above scaling relations for the SASA of the coil state and the globule state are based on qualitative arguments and are not necessary requirements for the applicability of the proposed mechanism. The proposed dependency of $\Delta G^{\mathrm{C}\to\mathrm{G}}_{\mathrm{Excl-Vol}}$ (and thereby the LCST) on the molecular weight holds true as long as the SASA of the coil state ($\mathrm{SASA}^{\mathrm{C}} \sim N^{\alpha_{\mathrm{C}}}$) grows faster with N than the globule state ($\mathrm{SASA}^{\mathrm{G}} \sim N^{\alpha_{\mathrm{G}}}$), $\alpha_{\mathrm{C}} > \alpha_{\mathrm{G}}$, which is true for all cases.

Supplementary Note 5

Effect of λ_{pa} on $\Delta G^{\mathrm{C}\to\mathrm{G}}_{\mathrm{Excl-Vol}}$

The solvent-excluded-volume contribution to the collapse free energy, $\Delta G^{\mathrm{C}\to\mathrm{G}}_{\mathrm{Excl-Vol}}$, has been obtained from thermodynamic integration calculations using the most probable coil and globule conformations. In these calculations, polymer-water and polymer-alcohol interactions were modeled with the purely repulsive Week-Chandler-Anderson (WCA) potential. Therefore, $\Delta G^{\mathrm{C}\to\mathrm{G}}_{\mathrm{Excl-Vol}}$ is determined only by the solute size and shape and the bulk solvent-cosolvent interactions. This can be seen from Fig. 10 where the dependence of $\Delta G^{\mathrm{C}\to\mathrm{G}}_{\mathrm{Excl-Vol}}$ on the methanol concentration for $\lambda_{\mathrm{pa}} = 0.849$ is same as that of $\lambda_{\mathrm{pa}} = 0.949$.

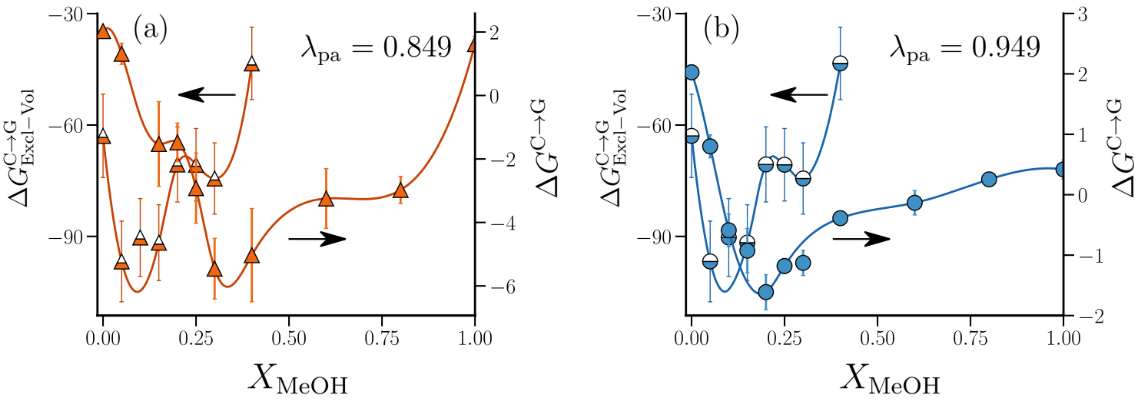

Supplementary Figure 10: Dependence of $\Delta G^{\mathrm{C}\to\mathrm{G}}_{\mathrm{Excl-Vol}}$ and $\Delta G^{\mathrm{C}\to\mathrm{G}}$ on the methanol concentration X_{MeOH} for (a) $\lambda_{\mathrm{pa}} = 0.849$ and (b) $\lambda_{\mathrm{pa}} = 0.949$. For both cases, $\lambda_{\mathrm{pw}} = 1.095$. The error bars represent the standard errors in the respective quantities.

References

1. Maffi, C., Baiesi, M., Casetti, L., Piazza, F. & De Los Rios, P. First-order coil-globule transition driven by vibrational entropy. *Nat. Commun.* **3**, 1065 (2012).

2. Tiktopulo, E. I. *et al.* “Domain” coil-globule transition in homopolymers. *Macromolecules* **28**, 7519–7524 (1995).

3. Palivec, V., Zadrazil, D. & Heyda, J. All-atom remd simulation of poly-N-isopropylacrylamide thermodynamics in water: a model with a distinct 2-state behavior. *arXiv* (2018). `1806.05592`.

4. Podewitz, M. *et al.* Coil-globule transition thermodynamics of poly(N-isopropylacrylamide). *J. Phys. Chem. B* **123**, 8838–8847 (2019).

5. Zangi, R., Zhou, R. & Berne, B. J. Urea’s action on hydrophobic interactions. *J. Am. Chem. Soc.* **131**, 1535–1541 (2009).

6. Dalgicdir, C. & Van der Vegt, N. F. A. Improved temperature behavior of PNIPAM in water with a modified OPLS model. *J. Phys. Chem. B* **123**, 3875–3883 (2019).

7. Dalgicdir, C., Rodríguez-Ropero, F. & Van der Vegt, N. F. A. Computational calorimetry of PNIPAM cononsolvency in water/methanol mixtures. *J. Phys. Chem. B* **121**, 7741–7748 (2017).

8. Kang, Y., Joo, H. & Kim, J. S. Collapse-swelling transitions of a thermoresponsive, single Poly(N-isopropylacrylamide) chain in water. *J. Phys. Chem. B* **120**, 13184–13192 (2016).

9. Garcìa, E. J. & Hasse, H. Studying equilibria of polymers in solution by direct molecular dynamics simulations: Poly(N-isopropylacrylamide) in water as a test case. *Eur. Phys. J.* **227**, 1547–1558 (2019).

10. Rodríguez-Ropero, F., Hajari, T. & Van der Vegt, N. F. A. Mechanism of polymer collapse in miscible good solvents. *J. Phys. Chem. B* **119**, 15780–15788 (2015).
